# Diversity of Intercellular Communication Modes: A Cancer Biology Perspective

**DOI:** 10.3390/cells13060495

**Published:** 2024-03-12

**Authors:** Thanzeela Ebrahim, Abdul Shukkur Ebrahim, Mustapha Kandouz

**Affiliations:** 1 Department of Pathology, Wayne State University School of Medicine, Detroit, MI 48202, USA; 2 Department of Ophthalmology, Visual and Anatomical Sciences, Wayne State University School of Medicine, Detroit, MI 48202, USA; 3 Karmanos Cancer Institute, Wayne State University School of Medicine, Detroit, MI 48202, USA

**Keywords:** intercellular communication, cancer, gap junction, tight junction, adherens junction, desmosome, exosome, extracellular vesicle, apoptotic bodies, eph, ephrin, bystander effect, tunelling nanotube

## Abstract

From the moment a cell is on the path to malignant transformation, its interaction with other cells from the microenvironment becomes altered. The flow of molecular information is at the heart of the cellular and systemic fate in tumors, and various processes participate in conveying key molecular information from or to certain cancer cells. For instance, the loss of tight junction molecules is part of the signal sent to cancer cells so that they are no longer bound to the primary tumors and are thus free to travel and metastasize. Upon the targeting of a single cell by a therapeutic drug, gap junctions are able to communicate death information to by-standing cells. The discovery of the importance of novel modes of cell–cell communication such as different types of extracellular vesicles or tunneling nanotubes is changing the way scientists look at these processes. However, are they all actively involved in different contexts at the same time or are they recruited to fulfill specific tasks? What does the multiplicity of modes mean for the overall progression of the disease? Here, we extend an open invitation to think about the overall significance of these questions, rather than engage in an elusive attempt at a systematic repertory of the mechanisms at play.

## 1. Introduction: Membrane-to-Membrane Communication and Cancer

It would be a tedious task to survey every single mode through which cells communicate with others, for intercellular interaction is key to the homeostatic life of multicellular communities. Nevertheless, one can still have a relatively global yet analytical overview of these modes in search of their similarities and peculiarities.

First, there is contactless communication that uses a limitless range of molecules such as growth factors and cytokines, that are free released by cells in their immediate microenvironment. Subsequently, these factors either act locally or are transported by various body fluids to remote areas. This mode of communication, probably the most classically studied, does not involve direct membrane-to-membrane interaction, and is not covered by this review, although it would be of utmost importance to know how contact-based communication differs from contactless communication and what is the functional significance of these differences.

**Figure 1:**
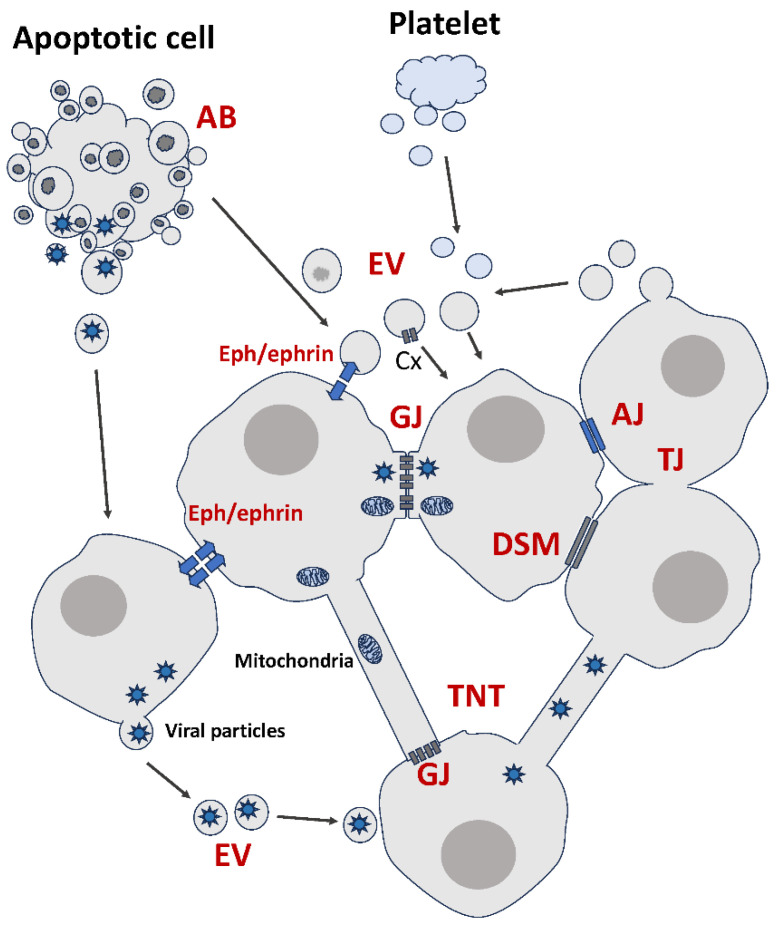
Summary of membrane-based modes of intercellular communication. AJ: Adherens Junction. GJ: Gap Junction. TJ: Tight Junction. DSM: Desmosome. EV: Extracellular Vesicle. AB: Apoptotic Body. TNT: Tunneling Nanotube. Cx: Connexin. See text for details.

Then, there is a growing repertoire of modes of communication that involve one form or another of membranous contacts between cells (Figure 1). After briefly introducing the distinctive features of each of these modes, we will discuss the significance and impact of the diversity of membranous intercellular communication, not through the lens of a systematic repertory, but rather by looking at functional aspects.

### 1.1. Junctions of All Kinds

Normal healthy cells are involved in complex organized multicellular tissues and are grouped into distinct tissular compartments endowed with specific functions. From that moment on, they become connected via different junctional structures. These specialized junctions, which allow intercellular communication and coordination, include gap junctions (GJs), tight junctions (TJs), adherens junctions (AJs) and desmosomes (DSMs). Each of these organizes into macromolecular complexes with structural and functional specificities. Based on their functions, cell junctions are classified into three groups. Communicating junctions, i.e., GJs, are involved in the exchange of molecules and electrical signals. Occluding junctions, i.e., TJs, prevent all molecular passage from cell to cell within epithelial tissues. Finally, structures responsible for cell–cell or cell–extracellular matrix (ECM) mechanical adherence are called anchoring junctions and include AJs, DSMs and focal adhesions (FAs).

#### 1.1.1. Gap Junctions

There is abundant literature that associates the different junctions to cancer. The role of gap junctional intercellular communications (GJICs) in cancer development and progression, as well as its potential as a therapeutic target, has been known for decades [1,2,3]. GJs are made of channels that couple the cytoplasm of two neighboring cells, thus allowing the passive diffusion exchange of small molecules [4,5,6]. GJs are also involved in electrical coupling and the synchronization of cells [7]. The major component of these structures are transmembrane proteins of the connexin family [8].

Among junctions, the particularity of GJs is their ability to form a direct channel between the cytoplasm of adjoined cells, whereby hydrophilic molecules can diffuse directly [9]. Another feature of interest is that the main component of GJs, namely connexins, has the ability to form two different types of structures: GJs and connexin hemichannels. GJs are formed by two opposing hemichannels each of which are contributed by the two cells engaged in the junction. Hemichannels are made of hexamers of connexins also called connexons. GJs are clustered into plaques with central pores [10].

In addition to ensuring metabolic coupling between cells, a distinctive feature of GJs is the ability to provide electrical conductance and coupling. Interestingly, many decades ago this function was shown to be absent in liver cancer cells while present in normal hepatocytes [11,12]. Indeed, GJs and connexins are essential for the biology of various healthy tissues, e.g., cardiac, vascular or neural, ensuring the propagation of electrical and chemical signals and cell synchronization. However, the significance of this function in cancer has received very little attention [13].

Another important feature of GJs that is not available in many other modes is the existence of a rapid biochemical control mechanism of its gating potential, through connexin post-translational modifications such as by phosphorylation [14,15].

#### 1.1.2. Tight Junctions

These structures are adhesive molecular complexes that are traditionally known for being important in preserving normal cellular integrity by establishing strong barriers. They are particularly essential for epithelial and endothelial tissues where they regulate cell polarity and paracellular permeability, respectively [16].

TJs are critical in cancer, particularly at the onset of metastasis. TJ loss affects tumor cell polarity, differentiation, adhesiveness, migration and invasiveness, all key steps in tumor metastasis [17,18,19,20].

Contrary to GJs, which function as movement facilitators, TJs are gatekeepers that tightly prevent intercellular molecular leakage and contribute to defining cellular structure and shape [16,21]. Understandably, the presence of TJs between cancer cells and neighboring cells, i.e., endothelial cells, impedes their efforts to undergo metastasis. TJ’s removal is thus a prerequisite for this process to occur [17,18,22,23,24,25]. However, TJs must not be regarded simply as idle stitches. While they bind to the cytoskeleton, they are also implicated in intracellular signaling [16], via components such as cytoplasmic adaptor proteins (e.g., zonula occludens (ZO)), or transmembrane linker proteins (e.g., occluding, claudins and junctional adhesion molecules (JAMs)) [19,21,26,27,28,29,30]. Claudins (CLDNs) for instance [31] have a signaling function that is bidirectional, sending signals to both cells involved in the interaction [19,32]. In this regard, they resemble junction-less proteins such as Eph/ephrins (see following sections).

#### 1.1.3. Adherens Junctions and Desmosomes

Along with TJs, AJs and DSMs are other types of junctions with importance in epithelial tissues. Both make use of cadherins and related proteins as cell adhesion molecules that connect between cytoskeletal structures between cells rather than constituting a membrane-to-membrane seal as found in TJs. Therefore, they both provide mechanical support to cells. However, AJs and DSMs differ in that DSMs are much more structured in line with their higher specialization. DSMs represent an increase in functional sophistication and effectiveness. They provide strength and mechanical resistance to tissues [33]. There is evidence to indicate a role of DSMs and protein components in cancer [34,35]. Similar to TJs, there seems to be a more prominent impact of AJs’ and DSMs’ disaggregation or loss in enhancing cell motility and metastasis, although their actual role in tumor suppression vs. oncogenesis might be context-dependent [36,37]. On the other hand, like TJs, AJs and DSMs are not mere structural scaffolds; they also have signaling functions that are important in the regulation of multiple cancer-related processes such as proliferation, apoptosis, differentiation and migration [35,38].

The adhesive and intercellular communication modes differ in their role in tumors, whether that be temporally, spatially or functionally. Even when structurally close, they still show important specificities. For instance, in vivo genetic experiments indicate that DSMs’ loss precedes that of AJs in the course of tumorigenesis and early invasion. Loss of DSMs weakens cell–cell adhesion, increases cell survival, promotes recruitment of inflammatory cells and increases local invasion. Subsequent loss of AJs adds to the loss of cell–cell adhesion and increases the extent of cellular invasion and distant metastasis [39]. Nevertheless, a major advantage of junction-based communication modes is their ability to regenerate and persist, while extracellular vesicles for instance have not been shown to be able to do the same (although it is possible to view restricted action as an advantage under certain circumstances).

Finally, it must be noted that although the above-mentioned intercellular junctions are the major ones featured in textbooks, there are many lesser-known junctions. It is reasonable to speculate that these could possess specialized functions in specific cancers, tumorigenic stages or conditions [40].

### 1.2. Junction-Less Proteins

Although proteins are the main components of the intercellular junctions, there are other protein categories whose role in cell–cell communication does not involve the formation of junctions per se. Many of these belong to the guidance proteins, so-called because they were initially discovered in the nervous system, where they control normal development and functions such as axon guidance, synapse formation and plasticity. Typical examples which will be featured here are the Erythropoietin-producing human hepatocellular carcinoma (Eph) receptors and their ligands called ephrins. Ephs and ephrins have a plethora of functions, prominently in the formation of spatial boundaries during embryonic development, skeletal development and angiogenesis [41,42,43,44,45,46,47,48,49,50,51,52,53,54,55].

Among the cell–cell communication modes, a first distinctive property of Eph receptors is that they are receptor tyrosine kinases (RTKs); in fact, the largest family of all. Thanks to an exceptionally large body of literature demonstrating the role of RTKs in cancer and their potential use as therapeutic targets [56,57,58], it is easy to understand the double importance of Ephs and ephrins as both RTKs and communication systems. Furthermore, unlike other RTKs such as growth factor receptors, including epidermal growth factor receptor (EGFR) and platelet-derived growth factor receptor (PDGFR), which are activated by free-released soluble ligands, both Eph receptors and cognate ephrin ligands are carried by the cell membranes of interacting cells. This leads to another particularity for this cell–cell communication mode: it elicits bidirectional signaling. A cell is acted upon at the same time that it acts on its interacting partner.

Functionally, in addition to their role in major cell homeostatic processes such as proliferation, cell death, invasion, etc., Ephs and ephrins also have the particularity of regulating cell positioning and sorting [47,59]. For instance, Eph and ephrin signaling regulates the positioning of intestinal epithelial cells within the stem cell niche [60], thus ensuring a coordination between intercellular communication, migration and cell positioning [61]. As mentioned earlier, Ephs and ephrins are guidance molecules first, that help direct cells through their microenvironment, balancing cell sorting and segregation on one hand, repulsion or attraction on the other hand. These properties endow invasive and metastatic cells with the potential to skip through the multiple boundaries between the primary tumor, the normal microenvironment and the endothelial system [62,63,64,65]. They also promote intermingling with normal cells of the metastatic niches [66,67].

### 1.3. Extracellular Vesicles

The idea that two cells that are remote could still undergo long distance membrane-to-membrane communications was not common knowledge before the discovery of extracellular vesicles (EVs). This generic name actually refers to a diverse population of membranous vesicles released by a plethora of cell types into the extracellular milieu. Depending on their origin and mode of genesis, different forms can be distinguished, mainly exosomes (<100–150 nm), microvesicles (<500–1000 nm) and apoptotic bodies (ABs) (<2–5 µm) [68,69,70,71]. Regardless of type, the biogenesis of EVs always involves the formation of lipid membrane-bound vesicles derived from endosomes or plasma membranes. This could be due to exocytosis of intracellular multivesicular bodies for exosomes, shedding the cytoplasmic membrane to form microvesicles, or fragmentation of the cell into membranous blebs in the case of apoptosis [72,73,74,75,76]. Therefore, EVs are small particles limited by phospholipid membranes that encapsulate cellular content from their cells of origin, and are released into the extracellular environment [77]. In fact, EVs are released by many cell types both alive or dying, from all organisms, both normal and pathological [78,79].

While their functions in multiple tumorigenic processes is steadily unravelling [80], EVs can be regarded as bona fide intercellular communication devices that receive and send signals [81,82,83,84,85,86,87,88,89,90,91,92,93,94]. Study of their role in tumor progression by virtue of sharing oncogenic molecules has been prominent in the recent literature [95,96,97,98,99,100,101]. Indeed, tumors can share oncogenic moieties using EVs. This is shown to involve their role in cell–cell communication between cancer cells and other cellular types in their microenvironment, whether normal or pathogenic. Examples include interactions with stromal cells, but also long distance with cells in the metastatic niches. EVs can be pro-angiogenic and participate in multiple steps during metastasis by eliciting local tumor invasion and ECM remodeling and epithelial–mesenchymal transition (EMT) [102,103]. Perhaps the most spectacular aspect of EVs in tumor biology is their role as emissaries from primary tumors to the metastatic niche, where they contribute to making these microenvironments more hospitable for secondary tumor growth [104].

Interestingly, dying cells are also able to communicate with other cells using a type of EVs [105,106]. Classic descriptions of apoptosis involve a sequence of events that includes nuclear chromatin condensation, nuclear fragmentation and cytoplasmic membrane blebbing. The apoptotic process culminates in the disassembly of the cellular content into multiple membrane-enclosed vesicles, known as apoptotic bodies (ABs) and normally destined to removal by phagocytes [107,108,109,110,111,112,113]. While exosomes and microvesicles are formed by healthy cells, ABs result from a cell death process reaching its end stage [70,79,114]. This leads to questioning the significance and impact of this communication from the cell’s death bed. There appears to be another distinctive feature for ABs, namely that they are more enriched in miRNAs than other EVs [115], although the significance of this finding is not clear. ABs are involved in the communication of dying cells with neighboring cells, particularly within the immune system [73,116,117,118,119,120]. ABs affect various functions including polarization, cell proliferation and differentiation [121,122,123]. However, it is not clear whether or not the fact that apoptosis-derived EVs inherit their content from terminally dying parent cells, rather than living cells, has any impact on their role in cancer.

Activated platelets are small cellular fragments. Nonetheless, they too can release EVs that reflect their molecular content and possibly also their features. Admittedly, the role of these EVs in cancer is not understood, but their amounts seem to be higher in cancer [124,125,126,127,128]. Although it is still speculative, there have been suggestions that these free-travelling platelet-derived EVs might bypass the limitations imparted upon activated parent platelets trapped in thrombi or other aggregates. This second-line messaging system would have the benefit of extending the platelets’ reach to heterologous cells beyond the blood vessels and circulating metastatic tumor cells, into tumor microenvironments and possibly even in the metastatic niche [129,130,131].

The above-mentioned EVs are not the only ones on record. Very large EVs have been identified, including large oncosomes, which are a distinct type of vesicle released by cancer cells [132,133,134,135,136]; exophers that transport protein aggregates and organelles [137]; and migrasomes that are released during migracytosis, a cell migration-dependent mechanism of vesicular secretion [138].

Regardless of the type or size of EVs, they all mediate an intercellular communication that combines the ability to undergo long-range effects along with the use of a membrane-based interaction. This allows EV-releasing cells to package multiple materials, even multiprotein complexes that would not otherwise be allowed through junctions. Instead of being delivered as single free released agents, multiple molecules could be delivered as molecular cocktails, in a targeted manner that relies on specific receptors. This mode also shields this cargo material from degradation and dilution [139]. Finally, as we have previously mentioned, unless proven otherwise, EVs seem to work as single-use packages, delivered with a time limit, and are unable to self-sustain or regenerate.

### 1.4. Tubular Structures

In relatively recent history, new modes of cell–cell communication in the form of protrusive nanotubular structures have been reported. These structures are very heterogenous and diverse and sometimes exist in the form of complex networks. Tunneling nanotubes (TNTs), which are tunnels with a diameter of 50 to 800 nm [140,141], are involved in the transfer of multiple types of cargo, including proteins, non-coding RNAs, calcium ions, nucleic acids, and even entire organelles such as mitochondria, lysosomes and autophagosomes [142,143,144,145,146,147,148,149,150,151]. A distinctive characteristic of TNTs is that the communicating cells engage in targeted membrane-based interaction without having to be in close proximity. It has also been reported that TNT-like structures appear to facilitate the transfer of membrane vesicles and organelles more than they do small molecules [140,148].

Shortly after its initial characterization, TNT-like communication was identified between cancer cells, or connecting cancer cells to intra-tumoral stromal cells [152,153,154,155,156,157,158,159]. TNTs have been shown to contribute to tumor cells’ adaptation and survival in response to stress, whether metabolic or other [155,160]. The fact that TNTs are involved in the transfer of functional mitochondria has led authors to propose that TNTs might be involved in the metabolic rescue of damaged apoptotic cells [161]. In other respect, TNTs between macrophages and breast tumor cells have been found to stimulate tumor cell invasiveness [162].

Because they seem to possess distinctive features in gliomas, TNT-like protrusive tunnels involved in the intercellular transfer of material have been given a different name: tumor microtubes (TMs) [163]. TMs are thicker and much longer than TNTs and form an extended network in gliomas [163,164,165]. In fact, there are two different types of TMs: the non-connecting open-ended type, and the interconnecting type which is associated with GJs [140,166,167]. Like other TNT-like structures, TMs have been shown to have a role in cancer, specifically in glioma progression [165,168], where they are at the fore of the invasive front, and associated with resistance to cell death induced by radiotherapy [163,168]. While both EVs and TNTs have the advantage of serving as cargo for large complexes, TNTs differ from EVs in that the former seem more targeted and directed.

## 2. Range of Communication

A key difference between the above-described modes of intercellular communication is the range and directionality of their action.

**Figure 2:**
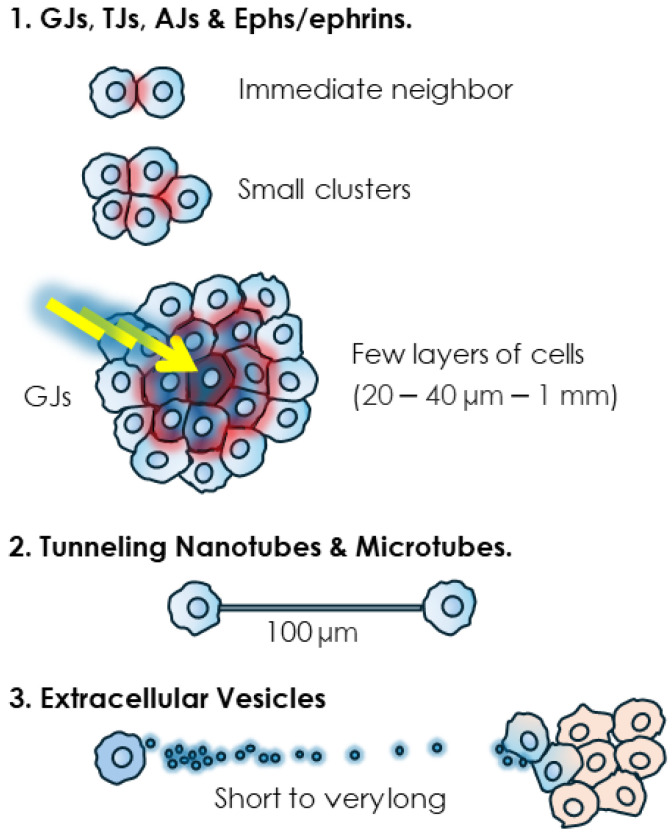
Range of action of different modes of intercellular communication. While various short-range junctions such as TJs and AJs, and junction-less Eph/ephrin proteins involve tight contacts, GJs are able, thanks to the “bystander effect” (BE), to transfer cytotoxic signals to multiple cells within few surrounding layers. Tunneling nanotubes (TNTs) and extracellular vesicles (EVs) extend this range of action significantly.

TJs, AJs, DSMs and the junction-less proteins such as Ephs and ephrins need direct and immediate cell–cell contacts (Figure 2). TJ’s short range of action is consistent with a role in local gatekeeping functions and the complex architecture more enmeshed with signaling complexes [169,170,171]. In the context of cancer, this locality in a sense forfeits reliance on the presence of masses of cells such as those seen in pre-cancerous lesions, primary tumors and established metastases. The reliance of TJs and their component proteins on cellular proximity, along with their multiplicity of roles (e.g., differentiation, cell polarity, proliferation, migration, stemness and EMT), gives them a functional flexibility that would explain a key role in metastasis [17,32], a process that involves a multitude of steps and functional changes. Interestingly, TJ proteins have been shown to have an important role in clusters of circulating tumor cells (CTCs) [172], which are tumor cells that have detached from the primary tumor and underwent the process of extravasation, as part of the metastatic journey [173,174]. Similarly, the expression of components of DSMs and AJs such as plakoglobin are significantly enriched in breast cancer CTC clusters which have a much higher (23–50-fold) metastatic potential than single CTCs [175]. Furthermore, plakoglobin knockdown blocks intercellular interactions, reducing CTC clustering and metastatic potential [175]. Traditionally, tumor cells were believed to metastasize as single cells entering the blood stream. However, since this model has been questioned, with increasing evidence of clustered metastatic cells [176], cell–cell interactions have subsequently gained more importance in metastasis. Indeed, the significance of this function of TJs is high, as this process of cellular aggregation contributes to the survival of metastatic cells by evading anchorage-dependent apoptosis (a.k.a. anoikis) [177]. It should be noted that an increasing number of cell–cell interaction proteins, not all featured herein, are identified that have a role in CTC clustering and metastatic potential. For instance, the cell surface glycoprotein intercellular adhesion molecule 1 (ICAM1, CD54) also promotes CTC cluster formation associated with a strong pro-metastatic role [178].

Junctional structures couple the cytoplasm of neighboring cells and in the case of GJs, this coupling can reach multiple cells from a single emitting cell. This ability to use GJs to share a signal, generally a cytotoxic one, with by-standing neighboring cells, is known as the “bystander effect” (BE) [1]. This phenomenon can thus extend the range of communication of the GJs to multiple cells. The determination of the maximum range of the BE is complicated by multiple factors such as dosage and concentration of the cytotoxic agent, cellular models, culture setting, as well as the presence or not of functional GJs. Furthermore, it is arduous to distinguish between the GJ-mediated transfer of cytotoxic agents vs. the transmission of their effects relayed by secondary metabolites or signaling molecules such as peroxides or oxidative stress-mediated signals. Nevertheless, early studies have provided a general idea, particularly pertaining to radiation-induced BE. Low doses of radiation particles, directly delivered to only 1% of Chinese hamster ovary (CHO) cells, inflicted a genome-damaging effect to up to 30% of non-irradiated cells [179]. In another experiment, irradiation of only 10% of confluent cells with a single α particle per cell resulted in the transmission of mutagenic effects to 100% of the cell population, an effect that could be prevented by blocking GJ-mediated BE [180]. It was estimated that the BE generated by α particles can propagate up to 1 mm away from the cells that are directly irradiated [181]. In a more targeted estimate, using the effect on stress-responsive protein p21^Waf1^ as a biological marker, it was found that low-dose α particle exposure induced a BE within a mean propagation distance ranging from 20 to 40 µm around the target, corresponding to approximately 30 cells [182]. While these differences in estimates could be due to the cell type and experimental set up and could also vary when cytotoxic stimuli other than radiation are considered, the conclusion is that GJs contribute to short and middle range cell–cell communication mainly within a cellular mass. This conclusion supports the privileged role for GJs and connexins in early-stage cancer promotion and primary tumors [1,183,184,185,186,187,188,189,190,191]. Admittedly, this role is likely to vary depending on cancer stages and, while the ability of GJs to propagate cytotoxic effects is in line with their tumor suppressive role, these structures also have tumor promoter roles [192,193,194].

As for tubular structures, they can couple the cytoplasm of significantly distant cell partners. Indeed, lengths of 6 µm [140] to 100 µm [195] to a few hundred micrometers [153,163], even exceeding 500 μm in astrocytomas [163,164,165], have been determined. TNTs are longer in sub-confluent cultures. It was shown that their length decreases as mesothelioma cells proliferate and the space between them diminishes to reach full confluence in vitro, thus possibly making this mode of communication unnecessary [152,153]. The invasive front, where other cell–cell interactions are dismantled, is also conducive to the formation and lengthening of TNT and TNT-like protrusions, as observed for instance when protrusions from astrocytoma cells extend and infiltrate the normal brain at the invasive front [163]. In the clinical setting, there is in fact a positive correlation of tubular length and unfavorable prognosis in gliomas [163].

In other respect, as will be further discussed below, TNTs can be functionally co-opted by GJs to provide a long-range GJIC, and some TNTs have functional GJ channels at their ends. For example, for TNT-mediated electrical coupling, the presence of GJs is necessary [167]. In addition to extending the range of GJIC, the TNT–GJ connection provides fine-tuning, in terms of electrical signal selectivity and amplitude [167].

EVs are abundantly found in vivo because they are transported by body fluids to remote environments. They have been detected in almost all body fluids including blood, urine, saliva, cerebral spinal fluid, amniotic fluid, breast milk, etc. [196]. They can thus be considered as the farthest-reaching mode of membrane-based intercellular communication.

## 3. Directionality of Molecular Transfer

Communication via TJs [169] and Ephs/ephrins [197,198,199,200,201,202,203,204,205] is bidirectional. For the latter, signaling bidirectionality has an impact on heterotypic cell–cell communication between cancer and normal cells [64,65]. The Eph/ephrin family has a large number of members that can be found in different combinations and expression patterns, as well as variable spatio-temporal dynamics [206], which provides this mode of communication with a high level of flexibility and diversity that is unavailable to other modes.

Tubular coupling opens the possibility of a bidirectional and specific exchange of material. For instance, it has been shown that in coculture, macrophages extend TNTs towards fibroblasts that are deficient in the lysosomal membrane cystine transporter, cystinosin. This results in the transfer of cystinosin-containing lysosomes into the deficient cells, which reciprocally use the same route to transfer cystine-containing lysosomes to the macrophages [207]. Whether similar exchanges are possible between cancer cells or between cancer and non-cancer cells awaits further examination. In an instance, a bidirectional transfer of vesicles, proteins and mitochondria was shown to occur in mesothelioma cells via TNTs, between two malignant cells or between two normal mesothelial cells, but not between a malignant and a normal cell [152]. In glioblastoma (GBM) cells, intercellular calcium waves are propagated bidirectionally via tumor microtubes [163].

Additionally, there is some evidence in favor of bidirectional communication via EVs, particularly in the context of the tumor microenvironment (TME), which is known for its cellular diversity and complex repertoire of heterotypic cell–cell interactions. Within the TME, cancer cells use EVs to communicate with normal stromal cells such as cancer-associated fibroblasts (CAFs) and vice versa. This EV-based exchange of material results in the activation of CAFs and reciprocally enhances the proliferation of tumor cells and metastatic potential [208]. The immune system is responsible for an essential anti-tumorigenic function, known as immunosurveillance [209]. Part of this function is mediated by EVs released by immune cells to target tumorigenic cells [104], which, in turn, use EVs to impact the TME [210]. EVs released and exchanged by both tumor-associated neutrophils and tumor cells collaborate to act on cells within the TME. The ensuing bidirectional communication is key in deciding the final pro-inflammatory and immunosuppressive response of these cells [211]. Furthermore, bidirectional communication via EVs between cancer stem cells and the microenvironment seems to be widespread in many solid cancers including prostate, breast, lung and colon [212]. Nevertheless, in some cases, EV-mediated communication was shown to be unidirectional. For example, transfer of miRNA via exosomes between T cells and antigen-presenting cells (APCs) during immune synapsis was shown to be unidirectional [213]. Cancer stem cells (CSCs) and mesenchymal stem cells (MSCs) are important cellular entities of the TME, both of which are sources of EVs, acting in a multidirectional manner in concert with tumor cells [214]. Tumor cells in hematological malignancies secrete EVs that engage in both unidirectional and bidirectional interactions with other cells of their TME, i.e., CSCs, MSCs, in addition to hematopoietic stem cells (HSCs). These interactions contribute to evading immune antitumor functions and developing therapeutic resistance [215].

As a final note, while it is expected that communication directionality would be of no concern, due to the unidirectionality of flow in the vascular and lymphatic systems, it would be interesting to examine how the ability of EVs to perform a long-distance action impacts the strength and specificity of the signal.

## 4. Transfer of Viral Particles

Viruses use a multitude of ways to spread from one cell to another [216]. Cancer etiology that involves oncogenic viruses gains a completely new dimension with the discovery of a vesicular transfer of viral particles. Not only is EV biogenesis mechanistically close to that of viral particles [217], it is also known that upon viral infection, cells release specific EV populations with distinct molecular repertoires [218]. Many oncogenic molecules, including Latent membrane protein 1 (LMP1), the major viral oncogene expressed in tumors associated with Epstein–Barr virus (EBV), are present in EVs released by infected cancer cells [219,220,221,222]. They are responsible for activating various signaling pathways, e.g., Akt and ERK, in uninfected target cells [222,223]. EVs’ contribution to virus-associated cancers also involves making changes to the TME [223,224]. EVs are not the only vesicular vehicle used by oncoviruses. It is intriguing that apoptotic bodies derived from infected cells can contribute to virus dissemination [225,226,227], thus posing the question of whether, unlike other modes of communication, EVs can increase the reach of viral oncogenes [123] (Figure 1).

EV and AB-mediated modes of transfer of viral particles are different from the so-called Retrovirus-like Particles (RLPs). These are isolated from various tumor cells both in vitro and in vivo [228,229,230,231,232]. RLPs are similar to retroviral vesicles in the sense that they originate from the budding of the plasma membrane [233] and contain some of the retroviral proteins [234,235,236,237,238,239], but they lack infectivity [111]. Nevertheless, although the use of vesicular communication for viral transfer can appear reminiscent of the natural mechanism of viral particle formation and release, other modes of cell–cell communication are also known to contribute.

Intercellular transfer of the tumor virus human T-cell lymphotropic virus type 1 (HTLV-1) involves both TJs and tubular structures (a.k.a. cellular conduits), although the significance of the latter mode is not clear [240,241,242]. Similar data support TNT-mediated viral transfer, whether in influenza [243,244] or AIDS [245,246,247,248]. In spite of these and other data showing the role of TNTs in the long-distance spread of viral particles [249,250,251], it is not known whether the ability of TNTs and similar structures to transfer viral particles, proteins and genomes contributes to tumor formation and/or progression.

Last but not least, rather than using GJs as a mode of transfer, many oncogenic viruses including human papillomavirus (HPV), simian virus 40 (SV40) and avian sarcoma virus impair GJIC mainly by decreasing expression levels [252,253,254,255,256], altering trafficking and recycling to the membrane [257] of connexin-43 (Cx43). This effect is in accord with the fact that cancerous cells often lose their ability to communicate via GJIC [1].

## 5. Transfer of Organelles

The role of organelles, particularly mitochondria, in cancer progression and therapeutic response is well established [258,259,260,261,262]. In addition to their essential role in intracellular metabolism, intact or fragmented mitochondria have been found in the extracellular milieu [263,264]. Mitochondria sharing has multiple roles in a multicellular communication context. Healthy mitochondria, for instance, can fuse with and rescue damaged mitochondria in neighboring cells and rescue the cell itself from apoptosis [161,265]. In addition to existing as free intact organelles or fragments (e.g., proteins, lipids, mitochondrial DNA), mitochondria can also be shared between cancer and non-cancerous cells via different communication modes, including GJs and TNTs [140,266,267,268,269,270]. Mitochondria can also be transferred via EVs [271,272,273] (Figure 1).

Considering the metabolic and energetic functions of mitochondria, and the importance of metabolism in cancer [274,275], it is normal that mitochondrial transfer would contribute to tumorigenesis by changing the bioenergetic and metabolic state of receiving cells. For instance, it has been shown that mitochondria transfer from astrocytes to cancer cells is prevalent and involved in GBM [276]. This transfer is dependent on a network of intercellular communications between GBM cells and astrocytes [276]. Furthermore, this astrocyte-to-GBM cells organelle transfer drives an increase in mitochondrial respiration and upregulation of metabolic pathways, thus promoting cell proliferation and tumorigenicity [276]. This mechanism depends on a protein called growth-associated protein 43 (GAP43), a major component of tumor microtubes (MTs) [276], a TNT-like tubular structure so far only found in glioma tumors including GBMs.

It is noticeable that the main route of mitochondrial transfer seems to be TNTs and EVs. Conceptually, it is understandable why, unlike many other modes of communication, vesicular and tubular modes would be structurally more amenable to organelle transfer, and this could prove to be a major, functionally relevant distinction between modes of cell–cell communication. This is not to say that there is no link between other modes of communication and mitochondria or metabolic functions. Positioning and intracellular trafficking of organelles has an essential role in cellular polarity [277,278], a process that is governed by TJs. In addition, targeting mitochondria’s function in the oxidative equilibrium has an impact on TJs’ integrity and function [279,280]. However, a role for junction-based interactions other than GJs in mitochondrial transfer has yet to be demonstrated.

## 6. Physical Constraints and Mechanical Communication

The existence of flexible cell–cell communication entities and others constrained in space, raises an intriguing question. Would physical and mechanical tensions have a different impact on the functions of different cell–cell communication modes, and vice versa? Biological processes involved in virtually all developmental stages and all physiological processes involve a level of mechanical constraints, whether between cells/tissues, or with ECMs [281,282,283]. This is certainly true in the context of tumor progression [284,285,286,287,288]. Cancer progression is associated with defects in mechanotransduction, associated with the ECM, focal adhesions (FAs) and cytoskeletal tensions [289,290,291].

### 6.1. Holding Tight, Sensing External Forces

Multiple structures such as membrane-anchored receptors, FAs, AJs or DSMs, constitute major sensors and transmitters of extracellular mechanical forces inside the cell, establishing a link more specifically with the actin cytoskeleton [292,293]. This process is known as mechanotransduction, which subsequently translates this force into many biochemical signal transduction pathways and ultimately impacts biological functions [294]. Mechanotransduction is essential for the understanding of both normal and pathological development [292,295,296,297].

GJIC and connexins are involved in the mechano-regulation of the functions of tendon cells, the tenocytes, in response to mechanical forces imposed on the tendons [298,299,300]. Connexins and GJs also help osteocytes communicate beyond the mechanical constraints of the bone minerals and transmit mechanical stimulation signals into the bone [301,302,303,304,305]. The mechanical pressure of laminar shear stress, due to blood flow and the cardiac cycle, increases the expression of Cx43, which facilitates the endothelial-to-mesenchymal transition of endothelial cells, a process involved in cardiovascular diseases [306].

Surprisingly, despite this evidence found in normal tissues, the current literature provides only limited data about the role of GJs or connexins in mechanotransduction in cancer. Numerous studies have shown that endothelial connexins and GJs modulate angiogenesis, including in tumors, by regulating several aspects of cellular mechanics, including endothelial tube formation, cellular stiffness and shear stress [307,308,309,310,311]. In addition, earlier studies have shown that it was possible to mechanically induce the release of intracellular calcium in normal and cancerous cells, possibly by affecting GJIC [312,313,314]. Another way GJs could affect the mechanobiology of cancer cells is through the driving flow of ions and water, thus regulating cell volume and osmotic pressure in proliferating cancer cells [315]. Cx43 participates in cytoskeletal dynamics by binding with microtubules and actin [316,317,318,319,320]. When Cx43 expression is downregulated in colorectal cancer cells, cell stiffness is reduced and stemness is increased, ultimately resulting in increased drug resistance [321]. Increased stiffness, in connection with the cytoskeleton, is a feature of tumors that is under control from both cell–cell and cell–ECM interactions [290,322].

### 6.2. Responding to the Microenvironment

**Figure 3:**
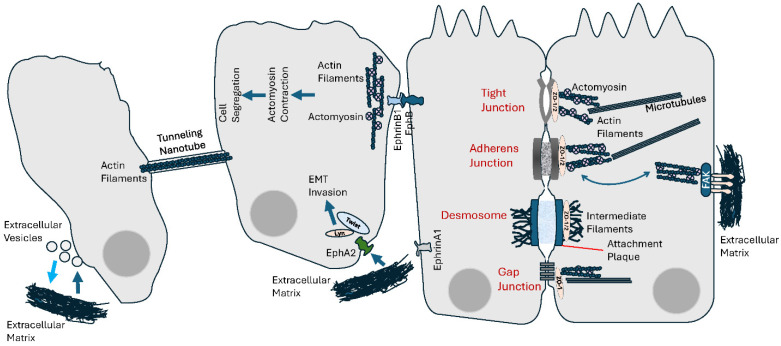
Cell–cell and cell–extracellular matrix (ECM) mechanical communication. Intercellular communication not only generates mechanical tensions, by interacting with the cytoskeleton, it is also responsive to endogenous mechanical forces as well as physical features of the ECM. See text for details.

There is evidence that EVs have a role in transmitting the mechanical tensions that contribute to their role in cancer, particularly at multiple levels during the metastatic process. Stiffness of the ECM affects EV cargo of miRNAs, a mechanism involved in modulating prostate cancer cell metastasis via regulating cell motility and ECM remodeling [323]. Liver cancer cells produce EVs when subject to fluid shear stress generated by the tumor microenvironment (TME) (Figure 3). These EVs have been shown to promote activation of normal fibroblasts into cancer-associated fibroblasts (CAFs), due to enrichment and activation in the IGF2-PI3K signaling pathway [324]. Similarly, exposure to the TME mechanical forces promotes invasive and pro-tumorigenic phenotypes in triple negative, but not ER-positive, breast cancer cells, a role that involves the immunomodulatory profile of the released EVs [325]. In other respects, physical forces have been shown to affect EV production (Figure 3). For instance, while cancer cells interact with the ECM, the latter’s stiffening also promotes the production of EVs by cancer cells which, therefore, also use cell–cell communication to contribute to tumor progression [326] (Figure 3). EVs were also found to transfer CKAP4, a protein associated with tumor metastasis in bladder cancer, and is responsible for the maintenance of a central-to-peripheral gradient of stiffness on the cell membrane. This EV-mediated transfer of CKAP4 enhances the migratory and metastatic potential of the recipient cells [327]. In addition to metastasis, EVs can also regulate cellular mechanoresponses during angiogenesis; under mechanical stress, fibroblasts’ secretion of EVs is enhanced, thus promoting angiogenesis [328]. EVs also have an impact on the physical environment of the bone metastatic niche, by regulating the production of proteins important for bone matrix stiffness and mechanotransduction [329]. The role of EVs in mechanotransduction is indirect, thus seems different than that of junctional structures which are intimately and mechanistically involved. The contribution of quantitative vs. qualitative changes in particular need to be further discriminated, as some of these effects could “simply” reflect overall profile changes within the cells of origin, rather than the specific and genuine changes and effects of EVs.

### 6.3. Spatial Positioning and Mechanical Communication

Together with other cell–cell interaction–regulatory proteins, Ephs/ephrins have critical functions during tissue development and organization, and morphogenesis [330]. The fact that Ephs/ephrins mediate cell–cell repulsion, cell and tissue segregation and boundary formation [331] is indicative of the extent of mechanical forces and tension at play [332]. Cell segregation driven by EphB/ephrinB1 signaling during tissue boundary formation is associated with actomyosin contractility, which generates a sustained mechanical cortical cytoskeleton tension [333,334] (Figure 3). In fact, the role of this signaling in cell segregation involves a decrease in the stability of heterotypic cell–cell contacts through increased cortical actomyosin contractility. In breast cancer cells, an EphA2/Lyn/Twist1 mechanosensitive signaling pathway has been identified in response to mechanical tension from the ECM (Figure 3). By inducing ligand-independent phosphorylation of the ephrin receptor EphA2, and subsequently recruiting Lyn/Twist1, ECM stiffness promotes EMT, cell invasion and metastasis [335]. The role of Ephs/ephrins involves an association with the mechanics of the plasma membrane as it connects its tension with the cytoskeletal machinery and signal transduction. Reciprocally, another particular feature of Ephs/ephrins is their bidirectionality, and it is very interesting to understand how mechanical forces could impact their spatiomechanical sensitivity. Spatial organization of Ephs/ephrins and sensitivity to mechanical influences are known to determine downstream signaling and cellular responses [336,337,338]. Spatial (re)organization of cell surface receptors and ligands under the influence of microenvironmental mechanical forces can determine the outcome of signal transduction [339,340,341,342]. The use of physical barriers to interfere with EphA2 receptor-ephrinA1 ligand binding, clustering and subsequent lateral transport results in changes to the cellular response of EphA2 to ephrinA1 in cancer cells [336] (Figure 3).

### 6.4. Collective Cell Migration

An example where mechanotransduction shows importance is the collective invasion of cancer cells into the surrounding non-cancerous tissue [343]. Migrating groups of cancer cells act as a cohesive and coordinated group, in which the link between and contraction of junctional cadherins and actomyosin cytoskeleton generates tension and physical forces [344]. Cancer-associated fibroblasts (CAFs), whose role in tumor invasion and metastasis is established, impose a physical force on cancer cells that enables their collective invasion [345]. Interestingly, many of the major communication structures involved, namely FAs, AJs and DSMs, use cadherins, whose role in mechanotransduction is well acknowledged [296,346,347]. There is also a relevant functional connection between FAs and AJs [347]. Mechanotransduction is sensitive to the nature of the cytoskeletal network’s anchorage sites. In other words, association with the actin cytoskeleton mediates the interplay between AJs and FAs in establishing a mechanical equilibrium between AJ-mediated cell–cell vs. FA-mediated cell–ECM interactions [296,347,348,349,350] (Figure 3). Reciprocally, the mechanical forces imposed on cells, particularly in the endothelium, affect junction formation [293,351,352,353].

The cellular group dynamic during migration can not only generate mechanical tension, it also affects other aspects as well. During collective cell migration (CCM), cells behave as either front leaders or rear followers. This structure is maintained by a polarized intercellular exchange of biochemical and mechanical information [354]. Due to their connection with the cytoskeleton, AJs and TJs are instrumental in establishing and maintaining this polarization during cell migration [355,356,357,358,359]. There is some evidence that the GJ connexin proteins might have some role in CCM, as their ability to transfer second messengers is crucial, at least in normal tissue such as during cardiac neural crest migration [360], wound closure [361] or feather elongation [362]. However, in cancer, evidence is not as abundant. GJIC and Cx43 have a role in facilitating collective migration in GBMs [363]. Another work showed that Cx43 hemichannels, but not GJIC, induce leader cell activity and collective migration of breast cancer cells [364]. Similarly, Ephs/ephrins seem to have a role in collective migration during thymus organogenesis [365] and directional collective cell migration of Schwann cells to help with peripheral nerve regeneration [366]. However, it is not clear if this cell–cell communication mode has any role in the CCM of cancer cells. The same could be said about EVs or TNTs, as the evidence is lacking in favor of their possible mechanistic involvement in or response to the collective dynamic of cellular migration.

## 7. Anti-Tumor Immunity

**Figure 4:**
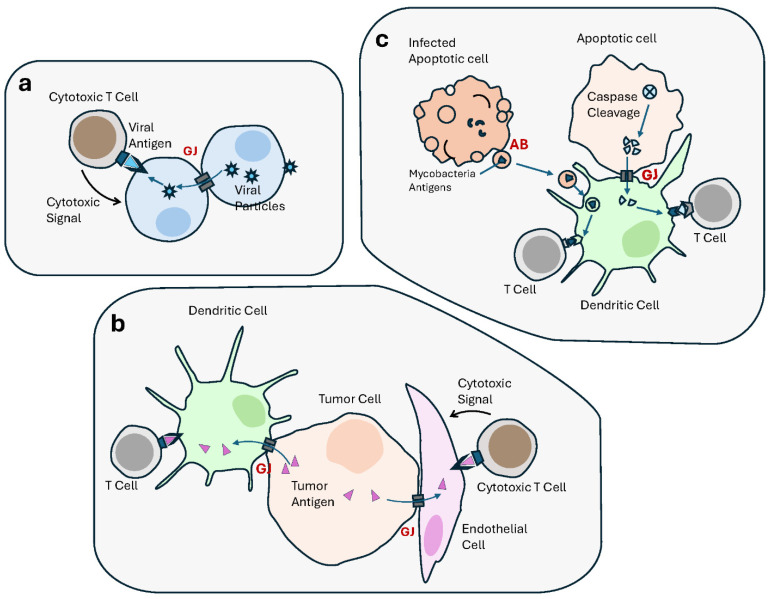
Cell–cell communication contributes to the immune responses, including anti-tumor immunity. Anti-tumor immunological defense mechanisms, including antigen (Ag) presentation, involve multiple modes of intercellular communication. (**a**) Gap junctions (GJs) transfer antigenic viral peptides from infected cells into noninfected neighbors, resulting in the recognition and targeting by cytotoxic T lymphocyte (CTL). (**b**) GJs allow Ag transfer from cancer cells to dendritic cells (DCs), which is presented to T Cells, as part of an anti-tumor immune response. GJs also transfer antigenic peptides between tumor cells and endothelial cells, resulting in the latter’s recognition and elimination by cytotoxic T lymphocytes. (**c**) In apoptotic tumor cells, GJs transfer antigens generated by caspase cleavage, from apoptotic cells to DCs, to be presented to T Cells, thus activating an antitumor immune response. Similarly, apoptotic bodies derived from apoptotic mycobacteria-infected macrophages contain mycobacteria-derived antigens which are transferred to DCs and presented to T Cells.

The establishment and function of host anti-tumor immunological defense mechanisms, including antigen (Ag) presentation, is part of the elements determining the fate of any developing malignant state. In spite of the long-standing interest in GJs, their contribution to these immune mechanisms has been largely understudied [367]. GJs allow antigenic viral peptides to be transferred from infected cells into by-standing noninfected neighbors, eliciting recognition by cytotoxic T lymphocyte (CTL) [367] (Figure 4). The advantage herein provided by GJIC to the immune defense mechanisms, as suggested by Neijssen et al., is that the elimination of bystanders mitigates risks of viral propagation and tumorigenesis [367,368]. In this regard, it would be understandable that the loss of GJs and connexins commonly observed in tumors [1,9,369] would be favorable to eluding anti-tumor immune defense mechanisms [370,371,372]. This hypothesis, nevertheless, awaits thorough exploration. In tumors, GJIC activity and connexin have been assigned both tumor suppression and tumor promotion functions [373]. Part of the reason behind this duality appears to be associated with their role in tumor immunity and interaction between tumor cells and stromal immune cells [374]. Evidence shows GJs as a way of communication, in particular with dendritic cells (DCs). For instance, GJs allow Ag transfer from melanoma to DCs, the latter subsequently being able to elicit a tumor-specific immune response in vivo [375,376]. GJICs are required for the activation of bone marrow-derived DCs [377]. In tumor cells, GJs are also involved in the transfer of antigenic peptides between tumor cells and the microenvironmental endothelial cells, leading to the latter’s recognition and elimination by cytotoxic T lymphocytes [378]. It has also been shown that GJs mediate the transfer of Ags produced by caspase cleavage, from apoptotic cells to be cross-presented by neighboring healthy and dendritic cells [379].

There is a recent interest in the roles of EVs in immune processes, such as inflammation and antigen presentation [380,381], or in cancer progression and immunotherapy [382,383,384,385,386]. Data support a role for EVs in modulating innate immunity both positively and negatively [387]. EVs help connect tumor cells with the immune system, coagulation, parenchymal tissue remodeling [388,389,390,391] and transfer immunosuppressive molecules including cytokines such as IL-10 and TGF-β1 as well as death receptor ligands such as FasL or TRAIL [392,393]. These immune functions also concern apoptotic cells, which have attracted attention as contributors to the immune response, e.g., Ag processing and presentation, including through their release of ABs and EVs [394,395]. EVs derived from mycobacteria-infected macrophages that undergo apoptosis transfer mycobacterial Ags to dendritic cells that drive their phagocytosis [396,397] (Figure 4). While the GJ-mediated delivery of antigenic moieties necessitates direct contact, tumor-derived EVs are released in various body fluids, to remotely reach Ag-presenting cells (including DCs). The latter take in the EVs and present the Ags on MHC I molecules to T cells, thus activating an antitumor immune response [89,398,399,400]. Nevertheless, like GJs, EVs can also contribute to escaping anti-tumor immune defense mechanisms, for instance by “flooding” the natural killer (NK) cells’ receptor NKG2D with EV-transported ligands, which results in receptor downregulation and impairment of the cytotoxic function of the immune cells [401], or by transporting Fas ligands (FasL) to trigger Fas-dependent apoptosis [402,403,404]. Innate immunity is an instrumental barricade in the face of tumor progression.

Data support a role of TNTs in immunity [405,406,407]. It has been shown that B-cell precursor acute lymphoblastic leukemia cells use TNTs to signal primary mesenchymal stromal cells (MSCs) to release pro-survival cytokines within their bone marrow microenvironment [408]. In general, it is too early to extensively discuss the burgeoning data regarding the connection between TNTs and anti-tumor immune mechanisms.

Last but not least, Ephs and ephrins have immunological functions including in tumor immunity [409,410,411]. Historically, the discovery of their specific role in vascular development and angiogenesis was tightly associated with the nature and mechanisms of their function [412]. For instance, the interaction between the EphB4 receptor on tumor cell surface and the cognate ephrin-B2 ligand on endothelial cells stimulates the latter’s invasion, survival and proliferation, and ultimately angiogenesis and tumor growth [413]. In addition, there is a plethora of evidence involving this large family of RTKs in endothelial activation, monocyte activation, adhesion, transmigration [414,415,416] and T-cell development and activation [417,418,419,420], among other processes of importance in immune responses. Ephs and ephrins contribute to tumor immune evasion by directly interacting with cells within the TME. The Eph/ephrin interaction is involved in regulating both innate and adaptive immunity [421]. Indeed, it was shown in head and neck squamous cell carcinoma (HNSCC) that the interaction between the EphB4 receptor and ephrin–B2 ligand, on the membranes of tumor cells interacting with their immune neighbors, affects the numbers of intratumoral immunosuppressive regulatory T cells (Tregs) [421].

## 8. Diversity and Heterogeneity

Tumor heterogeneity is instrumental in cancer progression and therapeutic response [422]. The coexistence, within the same tumor, of cells endowed with differences in their profiles of gene expression and genetic mutations is behind the possibility that, even under the most tumor-hostile conditions, a few cells have the potential to survive, metastasize or resist therapy. In addition to intra-tumoral heterogeneity, inter-tumoral heterogeneity renders cancer prognostic and therapeutic efforts even more complicated [423]. How intercellular communication factors into tumor heterogeneity is still largely unknown. In an attempt to explain the origins of heterogeneity and plasticity in melanomas, a model was proposed in which direct intercellular communication of tumor cells with endothelial cells is established in a perivascular niche, which results in the identity switch of a subset of stem-like cells that initiate metastases, independently from stimulation of tumor growth [424]. One can expect that any mode of communication that shows a large level of diversity and flexibility could contribute to this phenomenon. In this regard, two modes could provide such attributes and thus prove of particular interest: EVs and Ephs/ephrins.

A specificity of EVs in comparison with other modes of intercellular communication is their own diversity and heterogeneity [425,426,427,428,429,430]. Not only do they carry a plethora of molecular types, but both their physical characteristics, contents and amounts produced by tumors and other cells are also very variable depending on a multitude of factors, including cells of origin and physiological state. A proteomic profiling of EVs has found that they can differentiate between breast cancer molecular subtypes to an extent that is better than the whole cellular proteome itself [431]. In other respect, since the TME is important for intra-tumoral heterogeneity [432,433,434], communication of cancer cells with various cellular components of the TME using EVs could indicate the latter’s contribution to the process.

GJs and Eph/ephrins have a commonality that allows them to show a large level of diversity. Indeed, both the GJ connexins [435,436] and Ephs/ephrins [437,438] enlist many family members. All tissues express more than one type of these proteins, thus allowing them to form a huge number of combinations of homomeric or heteromeric complexes and homotypic or heterotypic intercellular communications. The challenge therefore becomes to understand the biological significance and dynamic of establishment of such a plethora of combinations in various tissues, both in space and time. Indeed, different connexins can engage in different channels endowed with different gating properties and selective permeability, depending on the tissue and other factors [6,439,440,441,442]. These considerations have a critical bearing in cancer, for instance for the ability of tumor cells to establish GJICs with endothelial cells, to cross the endothelial barrier and metastasize [443,444,445,446,447,448]. Similarly, evidence has accumulated regarding the discriminatory role of Ephs/ephrins in metastasis [449,450,451]. The large number and combinatory nature of expression and interactions of Ephs/ephrins in various tissues and different tumor subtypes [438,452,453,454,455] is particularly favorable to a potential role in establishing and/or maintaining tumor heterogeneity. However, this can only be speculative at the moment, despite the publication of a finding of subtype-dependent frequency and exclusivity of mutations in Ephrins in gastric cancers [456]. Probably most importantly, it is our hypothesis that, thanks to their prominent role in the sorting, patterning and positioning of cells, Ephs and ephrins could regulate the group dynamic between distinct sub-clones that develop and co-exist within the same tumor. Indeed, subclonal diversification is key to heterogeneity [457,458,459,460,461] and the various clones within the tumor engage in interaction and competition [462,463,464].

The role of junction-based intercellular communications in tumor heterogeneity is less clear. Beyond the findings that junction-associated proteins such as connexins, cadherins or claudins are differentially expressed in different tumor subtypes [465,466,467,468], the importance of engaging in the junctional communications per se is not known. In GBM, brain cancers known for their extreme heterogeneity, exists a subpopulation of cells that express the GJ protein Cx43 and engage in GJIC with nontumorigenic astrocytes, an interaction that promotes GBM cells’ invasiveness [469,470]. A study found that interfering with this communication, by genetically eliminating Cx43 from astrocytes, counteracts the GBM invasive activity [471]. Similarly, genetic depletion of the TJ protein E-cadherin was shown to contribute to the gain by breast tumors of the ductal subtype (IDC) of molecular features of invasive lobular breast carcinoma (ILC) [472]. In both cases, the data indicate that GJs and TJs could have a role in specific cellular subsets, although whether this would ultimately contribute to tumor heterogeneity awaits further elucidation.

This being said, apart from clonal selection, it has been suggested that cancer stem cells (CSCs) could also contribute to tumor heterogeneity [473,474,475,476]. Therefore, intercellular communication could also have a role in tumor heterogeneity via a function in cellular stemness. In fact, there are many studies in favor of a role of GJs [477,478] and TJ proteins [479,480,481] in CSCs, and other studies are concerned with the impact of EVs secreted by CSCs on tumor growth, progression, immune surveillance, metastasis, drug resistance and disease relapse [482,483,484]. TNTs were found to transfer mitochondria between GBM stem cells [485]. There are also studies that show for instance that mesenchymal stem cells (MSC) use TNTs to transfer mitochondria to glioma stem cells (GSCs), a mechanism that enhances GSCs’ resistance to the GBM drug temozolomide (TMZ) [486]. Eph signaling was also reported to impact tumor cell dormancy and CSCs’ enrichment after chemotherapy [487]. However, intriguingly, there is little evidence, if any, formally establishing a causality link between any of these intercellular communication modes and development or maintenance of tumor heterogeneity via a function in cancer stemness.

Finally, the diversity of modes of intercellular communication and/or predominance of a specific mode or another might by itself contribute to tumor heterogeneity, if certain cancer cells or colonies are shown to have proclivity to communicate via specific mode(s) rather than others.

## 9. Integration of Multiple Communication Modes

**Figure 5:**
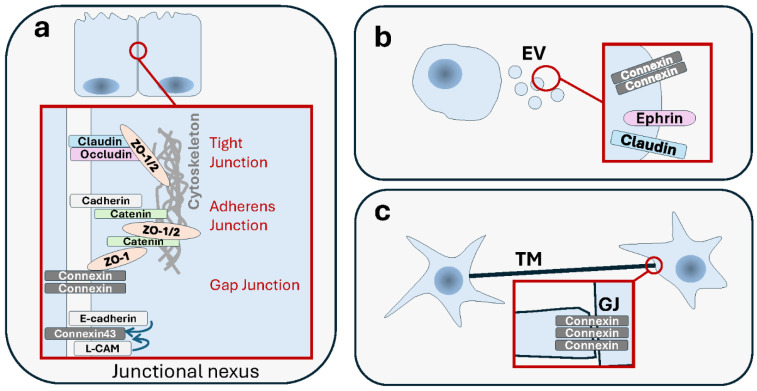
Interplay between various modes of cell–cell communication. Intercellular communication can be mediated by multiple modes at the same time, in a collaborative or antagonistic manner, depending on the context. (**a**) Junctional communication modes tight junctions (TJs) and adherens junctions (AJs) are structurally and functionally related and form a junctional complex. This “junctional nexus” interacts also with gap junctions (GJs). (**b**) Junctional proteins such as connexins or claudins, as well as Eph/ephrin proteins are transported remotely by extracellular vesicles (EVs), whose functions they contribute to. (**c**) Functional GJs are present in the ends of some TNTs and contribute to electrical coupling.

An essential aspect of understanding the diversity of cell–cell interaction modes is the study of their collaboration or opposition in various contexts, as well as their enrollment in complex meshes of communication. In this regard, evidence is available to show that modal interplays involve structural and functional aspects (Figure 5).

### 9.1. Junctional Nexus

The oldest established interplays involved junctions, such as the structural and functional relatedness between desmosomes and AJs, that gave rise to distinct specialized functions [488], or between AJs and TJs [489,490,491]. Connexins interact with many AJ and TJ proteins [492]. An early study has shown that the neural cell adhesion molecule (NCAM)-mediated cell–cell adhesion promotes GJIC, and that blockade of the former interferes with the latter [493]. E-cadherin and the cell–cell adhesion molecule L-CAM exert a control over GJIC, possibly via post-translational modification of Cx43 [494,495]. Reciprocally, overexpression of connexin-26 (Cx26) enhances cancer cell motility in a GJIC-dependent manner, and it also reduces cell adhesiveness and loss of N-cadherins [496]. Association between Cx43 and N-cadherin was shown to be required for the formation of both GJs and AJs [497]. Another study extends the interplay even further by showing the existence of a “junctional nexus” involving connexins along with TJs and AJs, and that is remodeled during mammary gland development [498] (Figure 5). These examples of connections could reflect context-specific functional differences or adaptation processes. When two epithelial cells engage in a new interaction, the changes that occur in the organization of the actin cytoskeleton following AJ formation are a prerequisite for subsequent TJ formation [499]. Genetic loss of DSM-mediated adhesion was shown to disturb TJs’ formation and barrier function in the skin [500], a tissue where AJs and TJs have been shown to be structurally interconnected [501]. In cancer, the interplay between these junctions needs to be seen through the prism of adaptation of tumor cells to the stress induced by loss of adhesion, remodeling of the TME, changes in the identity of interacting neighbors, and engaging in movements. The task is not easy if one needs to obtain not only descriptive data but, more importantly, functional evidence as well. Still, the data for now support that the interplay observed in normal settings is also observed in tumors. Importantly, cell–cell junctions are very often responsive to the same pathological processes, such as disruption in cell polarity and architecture [502]. In the normal mammary epithelial cells, proteins that control cell polarity regulate the establishment of different types of cell junctions together (TJs, AJs and GJs) along the basoapical axis, an association that is important in preventing tumorigenesis [503]. This shared fate has a mechanistic basis that is perturbed in cancer cells. For instance, progastrin, a prohormone whose plasma levels are high in patients with colorectal carcinoma, causes a concomitant dissociation of both TJs and AJs, but via two distinct pathways [504]. IQGAP1, a pro-oncogenic scaffolding protein involved in TJ establishment by impacting claudin localization, also inhibits AJ formation by sequestering E-cadherin from cell–cell contacts [505].

### 9.2. Cooperation for Long-Distance Action

More recently, evidence started to appear of an interplay between junction-based intercellular communication and EVs. There are data in vitro and in vivo that at least a major component of GJs, i.e., Cx43, is transported by EVs in the form of hexameric channels. Far from being a mere passenger, this Cx43 impacts the function of the supporting EVs. However, more evidence is needed to envisage that EV-born GJ channels are able to elicit GJIC in a manner similar to their cellular counterparts. If proven true, this would raise the question of the added value of using GJs to transfer material that is destined to be delivered via EVs anyway. A possibility is that what matters here is the delivery of GJ structures themselves, making this a mode of long distance and heterologous transfer of readily made GJs destined for rapid use by recipient cells [506]. In addition to EVs, GJs have a connection with TNTs [144,167]. Functional GJs were found in the ends of the membrane projections of TNTs [507]. A study showed that TNT-dependent electrical coupling depends on the presence of GJs [167]. In fact, a type of TNTs which accumulates Cx43 is able to couple cells electrically via GJIC [508].

Uptake by brain microvascular endothelial cells of EVs produced by neutrophils results in the dysregulation of expression of genes associated with TJs, thus increasing the endothelial cells’ permeability, and decreasing their transendothelial electrical resistance (TEER) [509]. EV-mediated transfer of TJ proteins claudin5 was proposed to facilitate leukocyte transendothelial migration (TEM) across the strong TJ-based obstacles part of the blood–brain barrier (BBB), by allowing temporary leukocyte/endothelial contacts [510]. Similarly, long noncoding RNA lnc-MMP2-2 in EVs promotes non-small cell lung cancer (NSCLC) brain metastasis, by dismantling TJs, increasing vascular permeability and compromising the integrity of the BBB [511]. The microRNA miR-105 transported by EVs released by breast cancer metastatic cells was shown to regulate TJs in endothelial cells by downregulating the TJ protein ZO-1, thus increasing vascular permeability, and facilitating metastasis [512].

Another noticeable integrated communication that awaits further investigation occurs between EVs and TNTs [513]. For instance, in mesothelioma cells, EVs were able to accelerate the rate of TNT formation [514]. Furthermore, both TNTs and EVs were concomitantly released by GBM cells under cocaine treatment [515]. Although no mechanistic link was established between the two events, this is in support of the existence of a multimodal and coordinated communication between EVs and TNTs.

Breaking with the classical view that Eph/ephrin interaction necessitates direct proximity of cells, with the occasional possibility of shedding of soluble molecular forms, it was reported that both Ephs and ephrins could be transported by EVs [516]. Osteoclasts release EVs that express ephrin-A2, which allows them the interaction with EphA2-bearing osteoblasts [517]. While these data have been found in the context of neural development and synapse physiology, as well as bone homeostasis, respectively, it would be important to examine whether similar mechanisms occur in cancer. So far, the data are scarce. EphA2 was found to be enriched in a specific subpopulation of EVs from various cancer cells [430]. It was found that EphB2 expressed in EVs that are released by head and neck squamous cell carcinoma cells regulates angiogenesis by stimulating ephrin-B reverse signaling in endothelial cells [518]. As rightly pointed out by the authors of this work, the existence of such a mode of Eph/ephrin long-distance interaction has the advantage of allowing hypoxic cells at the center of the tumor, to release EVs which exit the tumor and stimulate angiogenesis [518]. Senescent cells were shown to secrete EphA2-expressing EVs which promote cancer cells’ proliferation by binding to ephrin-A1 and inducing reverse signaling [519]. Furthermore, not only are EVs released by drug-resistant cancer cells enriched with EphA2, but they also transmit the invasive and metastatic phenotype from drug-resistant to sensitive cancer cells [520]. As much as these findings extend the range of action of Ephs and ephrins, it would also eventually dissociate between forward and reverse arms of the Eph/ephrin-mediated bidirectional signaling.

### 9.3. Ephs/Ephrins and Junctions: Conflict and Cooperation

Ephs/ephrins and cadherins coordinate their actions to regulate cell segregation and tissue boundary establishment [55,521,522,523]. In fact, evidence is tilted in favor of an effect of Ephs/ephrins on cadherin-based adhesion, despite the fact that there is evidence to support the reciprocal regulation of Eph/ephrin expression and function by cadherins [524,525]. For example, the role of Ephs/ephrins in establishing embryonic boundaries was shown to be mediated by an effect on cadherin-mediated adhesion [526]. Similarly, in the intestinal epithelium, the interaction between EphB receptors and E-cadherins at the interface with ephrin-B1-expressing cells, results in metalloproteinase-mediated cleavage of the E-cadherins and weakening of cell–cell adhesion [527]. EphA2 was reported to regulate TJ formation in brain endothelial cells [528]. Ephrin-A1 stimulation disrupts TJs and AJs, suggesting a role in the regulation of pulmonary vascular permeability [529]. EphB2 and ephrin-B1 expression is upregulated during the process of skin wound repair, resulting in the downregulation of AJ and TJ proteins and weakening of the adhesion between epidermal cells [530]. EphA4 activation results in the loss of AJs more than TJs [531]. Ephrin-B1 binds AJ component Pick1, and overexpression of the former phenocopies the loss of the latter, both resulting in the dissociation of cancer epithelial cells via disruption of the AJs [532]. EphA2 associates with and modulates the localization and function of TJ protein claudin-4, hindering the latter’s cell–cell adhesive function and increasing paracellular permeability [533]. A collaboration between EphA4 and metalloprotease ADAM10 in the cleavage of E-cadherin regulates the integrity of AJs between adjacent pillar cells in the cochlear sensory epithelium [534]. Ephrin-B1 associates physically with TJ proteins claudin-1 or claudin-4, promoting ephrin-B1 phosphorylation, and affecting cell–cell adhesion in cancer cells [535]. A positive feedback loop has even been proposed, in which E-cadherin-based junctions and EphA/ephrinA signaling enhance each other [536]. A difference between Eph/ephrin-mediated and E-cadherin-based interactions could be the propensity of the former to regulate heterotypic contacts at the interface of segregated cell populations, while the latter is more associated with homophilic contacts [537]. In addition to the above-mentioned examples, more evidence exists linking Ephs/ephrins and either TJs or AJs [538,539,540]. We have previously published an extensive survey of the interplay between Ephs/ephrins and junction-based communications in cancer [437].

Furthermore, there is a link between Ephs/ephrins and GJs. For example, Ephs/Ephrins regulate GJIC at the boundary of hindbrain cell populations [541]. Ephrin-B2 enhances the assembly of Cx30-based GJ plaques between non-sensory Deiters’ cells [542]. Mutations in Ephrin-B1 lead to human craniofrontonasal syndrome (CFNS), an effect mediated by changing Cx43 distribution and inhibition of GJIC at ectopic Ephrin boundaries [543]. Tampering with either Ephrin-B1 or Cx32/Cx34-based GJIC results in similar effects on the adhesion and dissociation of embryonic cells [544,545]. At least some of these effects involve physical interactions. EphB1 and EphA1 increase GJIC via phosphorylation of the Cx32 C-terminal domain [546]. There is an interaction between EphB4 and Cx43 in cardiomyocytes and EphB activation results in GJIC inhibition [547]. Interestingly, the possibility for Eph/ephrins to undergo bidirectional and unidirectional signaling provides a basis for discrimination between their bidirectional function in restricting cell intermingling versus their unidirectional role in GJIC [541].

## 10. Concluding Remarks and Perspectives

Aiming to draw a global overview of membrane-to-membrane-based modes of intercellular communication would have been a colossal task. That was not our aim here. Focusing on a few aspects and illustrative examples, we pointed out distinctive features worth considering in a comparative view. Whenever possible, we discussed the importance of these distinctions in cancer progression. However, some aspects, despite being conceptually important, were left out either because of scarcity of data (such as target determination), immense volume of data (such as the nature of molecular cargo, or signal transduction coupling), prematurity of the task (such as implications for therapeutic targeting) or simply because they have been amply reviewed in previous publications.

What determinants drive and what machinery regulates contact establishment during intercellular communication is an area of research that is amply understudied, particularly for the more recently discovered modes of interaction such as EVs and TNTs. For instance, the presence of specific proteins and peptides such as receptors and adhesion molecules has been involved in cell-specific targeting by EVs [548,549,550]. Various elements such as the cell of origin, the target cells’ identity or the physiological state contribute to this determinism [551]. However, on a larger scale, the mechanistic and biological determinants of EV targeting are yet to be explored.

While it is essential to compare the molecular cargo profile between various modes of communication, possible predilection for the transfer of certain molecular types or functional categories, extent of transfer of complexes, structural vs. bioactive cargo, speed and extent of transfer, etc., recording cargo content would be a daunting task in view of the still rapidly ongoing developments in this area. Suffice it to say that virtually all categories of molecules have been shown to be transferred between cells using these modes, whether different types of nucleic acids, proteins, peptides, ions, etc. Technological profiling tools can only provide part of the answer to the large number of lingering questions. For instance, proteomic analysis of EVs collected from the NCI-60 human tumor cell line panel showed that they reflect the proteomes of their cells of origin [552], and showed an association between the abundance of a subgroup of proteins and specific cancer hallmark signatures [553]. More large-scale methods can evidently provide large data sets. A starting point towards a higher order analysis of the differences and their functional significance would be the establishment of centralized databases (e.g., the EVs’ database ExoCarta: http://www.exocarta.org). Nevertheless, as for all tools of global repertory, new concepts need to be developed to make sense of the collected data. Let us take the case of nucleic acids to illustrate some aspects of the complexity of knowledge that needs to be made sense of. We know that GJs are able to transmit DNA moieties. Small fragments of DNA such as short interfering RNAs (siRNAs) or microRNAs can move via GJs from a target cell to a neighboring cell, where they can inhibit gene expression and even induce resistance of cancer cells to chemotherapy [554,555,556,557]. However, it appears that GJs’ ability to transfer genetic material is somehow limited, at least concerning siRNAs, as the rate of transfer decreases with increasing length [554]. Nevertheless, small RNA transfer through GJs seems to be more efficient than through EVs [555,558]. In addition to proteins, EVs transfer a large variety of nucleic acids, including coding RNAs (mRNAs) and noncoding RNAs (long noncoding RNAs, microRNAs and circular RNAs) [559,560,561]. EV-transferred noncoding RNAs for instance have a role in cancer immune escape [562]. The particular example of ABs also carry nucleic acids, but they differ from EVs in their RNA cargo, with the former being more enriched in rRNA [563]. EVs are able to transfer DNA horizontally between cells [564,565,566,567]. EVs contain microRNAs which contribute to cancer progression by modulating intercellular communication [568]. Furthermore, not only has DNA been detected in ABs upon apoptotic fragmentation [569,570], but it is also used by cells involved in ABs’ phagocytosis [570,571]. EVs can also function as secondary carriers of genetic material, by transferring mitochondria loaded with DNA [572,573]. In fact, mitochondria can release EVs in their own right [574,575,576,577,578]. Interestingly, while higher levels of mitochondrial DNA in the plasma have been associated with cancer [579], it has been shown that a portion of it is carried by EVs [580], and that it can contribute to therapeutic resistance of breast cancer cells [581]. DNA fragments found in the bloodstream, a fraction of which is of mitochondrial origin, are known as circulating free DNA (cfDNAs), and there is some evidence that most of the cfDNAs is contained in EVs [132,582,583]. This is of significance since this blood-circulating genetic material is believed to have an oncogenic potential [584,585,586,587,588,589].

Admittedly, this review does not provide an answer to the intriguing question of the most functionally instrumental difference between modes of communication that rely on chemical release interaction versus those depending on direct membrane-to-membrane interaction. The task is rendered more challenging by the shared features between the two types of modes. Among membrane-based cell–cell communication modes, EVs might be the closest to released single molecules such as growth factors, in that both act at a long distance, including via the bloodstream, thus bypassing the imperative of close proximity which is limited by tissular rigidity and cell density. A major difference, though, is the EVs’ ability to transfer molecular complexes and cocktails rather than single molecules. In other respects, soluble and functional forms of Ephs/ephrins have been reported as a result of cleavage by matrix metalloproteases (e.g., [590,591,592,593,594,595]). However, a key particularity of Ephs and Ephrins is that, unlike communication via growth factor receptors (e.g., EGFR and PDGFR), which are activated by free soluble ligands, both the Eph receptors and the ephrin ligands are membrane-bound and both initiate signaling into their respective cells, whether it is the forward signaling that originates from the Eph receptor or the reverse signaling that originates from the ligand. This is a tremendous difference, as this signaling involves two partner cells that could be of similar or different types or origin, as well as either cancerous or normal. This bidirectionality is also observed in junction-based communication and EVs. It is also noteworthy that another distinctive feature of Ephs/ephrins is that they constitute the largest subgroup of RTKs. This has a double impact. First, biochemically they behave quite like other non-membranous communication receptors such as EGFR or PDGFR and phosphorylation is essential for their roles in cancer [596,597,598,599,600]. Secondly, they too attracted therapeutic targeting attempts based on kinase inhibitors, with more or less success [601,602,603,604].

To end, a chronological sequence of genetic and biochemical events that target specific components of each junction throughout tumor progression has yet to be established, in order to further understand the dynamic of the interplay between the different modes. For instance, the role of junctional complexes, including TJs, as adhesive structures that ensure intercellular association appears, understandably, to be the logical first barrier that tumor cells need to silence in order to metastasize [17,18,605]. However, TJs occasionally have an opposite function by promoting EMT and tumorigenesis [20]. In conclusion, an effort to obtain a global overview of these events is needed, that reaches beyond the critical biochemical and structural commonalities and interconnections, to elucidate a higher order functional significance.
